# Corrigendum to: 100 years post-insulin: immunotherapy as the next frontier in type 1 diabetes

**DOI:** 10.1093/immadv/ltac013

**Published:** 2022-06-22

James A Pearson, Eoin F McKinney, Lucy S K Walker, 100 years post-insulin: immunotherapy as the next frontier in type 1 diabetes, *Immunotherapy Advances*, Volume 1, Issue 1, January 2021, ltab024, https://doi.org/10.1093/immadv/ltab024

In the originally published version of this manuscript, several in-text citation numbers were incorrect and pointed to the wrong references.

These errors have been corrected.

